# The associated factors for physical activity-related injuries among first-year university students in southern China from a biopsychosocial perspective

**DOI:** 10.3389/fpubh.2024.1369583

**Published:** 2024-04-02

**Authors:** Lingyu Xu, Shangmin Chen, Dongna Gao, Ying Fang, Liping Li

**Affiliations:** ^1^School of Public Health, Shantou University, Shantou, China; ^2^Injury Prevention Research Center, Shantou University Medical College, Shantou, China; ^3^Mental Health Center, Shantou University, Shantou, China

**Keywords:** physical activity-related injuries, physical activity, bio-psycho-social medical model, injury prevention, injury

## Abstract

**Background:**

Understanding the diverse factors influencing physical activity-related injuries is crucial for developing effective interventions that enable individuals to participate in physical activity (PA) while minimizing injury risk. Currently, research evidence on the multiple factors associated with PA-related injuries is inadequate. This study aimed to examine the associations between PA-related injuries and various biological, psychological, and social factors among first-year university students in China.

**Methods:**

We recruited first-year university students from Shantou University in Guangdong Province, China, to participate in our study. Data collection employed a structured self-administered questionnaire, gathering information on PA-related injuries, as well as relevant biological, psychological, and social factors. Binary logistic regression, using a stepwise modeling approach, was employed for the data analysis.

**Results:**

Among 1,051 first-year university students, 28.16% reported having experienced PA-related injuries in the past year. Most of the injuries reported were minor, with the knee or lower leg being the most frequently injured part of the body. Improper posture, environmental conditions, and excessive physical load were the leading causes of PA-related injuries. Multiple logistic regression analysis revealed that female students (OR = 0.67, 95% CI: 0.47–0.94, *p* = 0.022) had reduced odds of PA-related injuries. Conversely, high neuroticism (OR = 1.61, 95% CI: 1.07–2.41, *p* = 0.022), being a member of a sports team (OR = 2.09, 95% CI: 1.34–3.27, *p* < 0.001), PA on the wet ground (OR = 1.73, 95% CI: 1.18–2.54, *p* = 0.005) increased the odds of PA-related injuries.

**Conclusion:**

Our findings underscore the intricate interplay of various factors contributing to PA-related injuries. Identifying high-risk individuals based on physiological and psychological characteristics, coupled with targeted interventions addressing modifiable risk factors, is crucial for effective prevention.

## 1 Introduction

Physical inactivity contributes significantly to global noncommunicable disease prevalence and mortality (1). Recent global reports show that 27.5% of adults worldwide do not meet the recommended levels of physical activity (PA) (2). Insufficient levels of PA among adults constitute a global public health concern. Consequently, the World Health Organization (WHO) promotes public engagement in PA (3). Nonetheless, the potential adverse consequences of PA include injuries (4), and people need to pay attention to preventing PA-related injuries to ensure that they can engage in PA safely and consistently. Understanding the factors influencing PA-related injuries not only facilitates the development of effective interventions to reduce PA-related injuries but also contributes to the development of PA promotion strategies. By reducing the risk of injuries, these strategies can truly promote active public participation in PA. The acknowledgment of a multifaceted interplay among biological, psychological, and social factors has persisted for decades and was systematically elucidated by Engel, as the bio-psycho-social medical model (5), which is similarly applicable to PA-related injuries. The bio-psycho-social medical model is a comprehensive medical framework that aims to provide a comprehensive understanding of health and disease. The model emphasizes the interrelationship between biological, psychological, and social factors, arguing that each of these aspects plays a key role in influencing an individual’s health and disease.

In terms of biological factors, there is inconsistent research evidence on the association between gender and PA-related injuries. Some studies have suggested that men have a greater risk of PA-related injuries than women (6–11), while others have found that there is no significant difference in injury incidence or risk between the genders (12, 13). Similarly, there are inconsistent results on the association between chronic diseases and PA-related injuries (6, 14, 15). Furthermore, the biological factors associated with PA-related injuries include age (11, 14), BMI index (14, 16), allergy history (15), and so forth. In terms of psychological factors, anxiety, stress from negative life events, and daily hassles have been proven to be associated with PA-related injuries (17). In addition, some studies have suggested that students with high levels of antisocial and/or rebellious traits are more susceptible to PA-related injuries (18). Individual safety awareness also influences the occurrence of PA-related injuries (6, 14). In terms of social factors, several studies have found that there were differences in the incidence of PA-related injuries among students living in various geographical areas (6, 19). Notably, the PA environment significantly influences the occurrence of PA-related injuries, such as terrain, lighting conditions, and weather. Some studies have found that activities conducted on the wet/uneven floor, under poor lighting, or during extreme temperatures increased the occurrence of PA-related injuries (10, 14). Within the bio-psycho-social model, these environmental conditions are conceptualized as “social factors” due to their substantial impact on shaping social behaviors and indirectly affecting health outcomes by influencing individuals’ opportunities for social interaction and PA. Adverse environmental conditions can restrict outdoor activities, affecting social participation and PA levels, thereby influencing the health of individuals. Furthermore, some studies indicated that certain groups, such as first-year university students and athletic students were more susceptible to PA-related injuries (7). Within the bio-psycho-social model, health behaviors can be considered social associated factors (20). Previous studies have shown that prolonged sleep duration (6, 7, 14, 21–23), extended periods of sedentary behavior (6, 9), and excessive screen time (6, 24) are related to PA-related injuries.

In recent years, there has been a growing body of research on the risk factors of PA-related injuries. However, many of these studies have primarily addressed specific aspects of PA (11, 25–28). Furthermore, some studies have been conducted primarily on athletes (15, 21, 23, 29, 30) and adolescents (8–12, 14, 16, 21–24, 26). Nevertheless, given the growing public health concern of PA inactivity among adults, investigating the associated factors of PA-related injuries among university students becomes particularly essential. This is because university students transition from the academic burdens of previous education, gain more freedom and independence, and have increased opportunities to participate in various forms of PA (4, 13, 31). These individuals are also in a crucial transition period for lifestyle and habits (32). Consequently, the early implementation of preventive measures for first-year university students has the potential to benefit them in adulthood (33, 34). Given these considerations, the present study aimed to explore the associated factors of PA-related injuries among first-year university students from a biopsychosocial perspective. Exploring factors across biological, psychological, and social aspects allows comprehensive risk assessments and the implementation of multiple risk factor interventions during the enrollment of first-year university students. Identifying immutable risk factors, such as gender and age, contributes to assessing PA-related injuries risk levels in different populations and identifying high-risk groups for PA-related injuries. Simultaneously, modifiable risk factors, such as environmental and health behaviors, can be targeted in PA-related injuries intervention measures to reduce the incidence of PA-related injuries.

## 2 Materials and methods

### 2.1 Study design and participants

This cross-sectional study was conducted in October 2021 at Shantou University in Guangdong, China. First-year students from 8 colleges of Shantou University were selected as participants using cluster random sampling. The sample size for our study was calculated using PASS 15 software, selecting the two-sided interval type with a confidence level of 0.95 and a confidence interval width of 0.06. Anticipating a proportion of 0.298 based on prior research and domain knowledge, the minimum required sample size was determined to be 924. Considering a 20% potential dropout rate, the required sample size was adjusted to 1,155 to ensure statistical robustness and enhance the study’s credibility. To implement our sampling strategy, we established a sampling frame using a comprehensive list of all colleges and their majors at Shantou University. Through simple random sampling, majors were chosen and every first-year student in the selected majors was surveyed during their compulsory entrance health examination at the university’s medical center. The survey was made accessible via a link or QR code for electronic questionnaires, enabling students to complete it on their smartphones while waiting for their health examination. Additionally, students completing the survey were eligible for a random amount of a digital cash gift as an incentive. Eligible participants were first-year undergraduates at Shantou University who provided informed consent and had no history of serious physical illness. Participants were excluded from the analysis if they had incomplete or missing data, unreliable values for the variables, insufficient time to complete the questionnaire, or were under the age of 18.

Data were collected using a structured self-administered questionnaire distributed through the Wenjuanxing questionnaire platform to gather information on PA-related injuries and relevant biological, psychological, and social factors. In our study, a self-administered questionnaire was designed by applying a biopsychosocial approach. Before participation, the students were fully informed of the study’s purpose, procedures, and the voluntary nature of their involvement. It was ensured that informed consent was obtained from all participants. Three trained researchers and one university medical center staff member assisted students in completing and submitting the questionnaires. Researchers reviewed survey data for completeness and logical consistency to ensure validity. The data were handled anonymously to protect the privacy of the students. In total, 1,119 students agreed to participate in this study, of whom 1,051 first-year students met the study criteria. We obtained ethical approval from the Shantou University Medical College Ethics Committee (SUMC-2020-65).

### 2.2 Measurements

#### 2.2.1 Measurement of PA-related injuries

Given the absence of a specific PA-related injuries questionnaire for general university students, we used standardized questions to collect information on PA-related injuries and related biological, psychological, and social factors. The term “PA-related injuries” is defined as various injuries that may occur suddenly or gradually in one or more body parts while engaging in any PA. These injuries must result in at least one of the following conditions (11, 35): (a) immediate cessation of the PA, (b) inability to attend the next scheduled PA, (c) absence from class the next day, or (d) the need for medical attention. All participants were required to report whether they had experienced PA-related injuries in the past year based on the criteria mentioned above, and the frequency of PA-related injuries incidents was recorded. Participants who had experienced PA-related injuries were also asked to provide detailed information about their most recent PA-related injuries incident, including the body parts injured, the type of injury, the cause of injury, the severity of the injury, and subsequent treatment received.

#### 2.2.2 Measurement of biological variables

We collected some biological information from the students’ physical examination reports, including their gender (male or female), age, weight, height, myopia status (yes or no), and the presence of chronic diseases (yes or no). The body mass index (BMI) was computed by dividing a person’s weight in kilograms by the square of their height in square meters. BMI was classified in accordance with Chinese standards recommended by Chinese health authorities, categorizing students as underweight (<18.5), normal weight (18.5–23.9), overweight (24–27.9), or obese (≥28) (36). Moreover, students provided subjective assessments of their physical fitness (poor or good) through the self-administered questionnaire. They also reported their frequency of engagement in PA during physical fatigue or discomfort, choosing from one of the following four options: “never,” “rarely,” “sometimes,” or “frequently.”

#### 2.2.3 Measurement of psychological variables

To evaluate students’ psychological characteristics, we utilized two widely recognized psychological assessment instruments: the modified Chinese version of the Symptom Checklist-90 (SCL-90) (37, 38) and the Eysenck Personality Questionnaire-Revised Short Scale for Chinese (EPQ-RSC) (39). The SCL-90, comprising 90 items categorized into 9 factors, has been widely utilized to assess various psychological symptoms among university students in China, consistently demonstrating strong reliability and validity (40). Participants were required to rate each item on a 5-point Likert scale (1 = none, 2 = mild, 3 = moderate, 4 = severe, 5 = extremely severe). Factor scores were calculated by dividing the total score of items within each specific factor by the number of items in that factor, and these scores were subsequently used to evaluate participants’ psychological symptoms or mental health according to established criteria. In this study, we exclusively analyzed the following 5 factors: anxiety, depression, hostility, obsessive/compulsive, and paranoid ideation. The EPQ-RSC consists of 88 items, and is an instrument used to assess personality traits such as neuroticism, extraversion, and psychoticism (41). Participants responded to a series of structured questions with “yes” or “no” answers. Subsequently, we calculated the scores for each subscale and then converted them to standardized scores (T scores) using the formula: T score = 50 + 10 * (X − M)/SD. After obtaining the T scores, we translated them into three discrete categories based on the assessment criteria. These psychological assessment instruments have undergone validation and reliability testing to ensure their effectiveness (39). Additionally, we inquired about participants’ self-protective awareness during PA through the self-administered questionnaire, with response options of poor, normal, and good.

#### 2.2.4 Measurement of social variables

The participants’ college affiliation with Shantou University was obtained from their health examination reports. Additionally, the structured self-administered questionnaire was utilized to inquire whether the student was a member of a sports team. These social characteristics are considered social factors in this study based on previous research suggesting potential associations between college affiliation, sports team membership, and PA-related injuries among university students (7). Our study specifically addresses the following health behavior variables: PA, sleep duration (<7 h/day, 7–9 h/day, ≥9 h/day), sedentary behavior (<4 h/day, 4–8 h/day, ≥8 h/day), screen time (<2 h/day, 2–4 h/day, ≥4 h/day), alcohol consumption (no, yes), and smoking (no, yes). In terms of PA, participants were asked to provide comprehensive information. We collected data on the average frequency of PA over the past 12 months (never, ≤2 times/week, 3–5 times/week, ≥6 times/week) and the average duration per session (≤30 min, 0.5–1 h, ≥1 h). Additionally, we inquired whether their PA levels met the recommended guidelines for adults (categorized as below, meet, above), which were based on the WHO PA guidelines. The WHO guidelines for adults stipulate that individuals should engage in at least 150–300 min per week of moderate-intensity aerobic activity, at least 75–150 min per week of vigorous-intensity aerobic activity, or an equivalent combination of moderate and vigorous-intensity activities (2). Regarding the environmental factors affecting PA-related injuries, the questionnaire included 7 items with response options of never, rarely, sometimes, and frequently. These questions encompassed the following aspects: (a) Participation in PA on the wet ground, (b) Participation in PA on the uneven floor, (c) Participation in PA under insufficient light, (d) Participation in PA during hot weather, (e) Participation in PA during cold weather, (f) Participation in PA during rainy weather, and (g) Participation in PA in crowded places.

### 2.3 Statistical analysis

Out of the initial 1,119 samples, 1,051 participants (93.92%) met the eligibility criteria for further analysis, considering both inclusion and exclusion criteria. Categorical variables were expressed as counts (percentages), while continuous variables were represented by means and standard deviations (SDs). This study aimed to explore the associations between PA-related injuries and biological, psychological, and social factors. All the independent variables were categorized into three groups: (a) biological variables, (b) psychological variables, and (c) social variables.

The analysis initially employed univariate logistic regression analysis, followed by the application of multiple logistic regression analysis. In the first step, potential associated factors for PA-related injuries were identified. Variables that did not show statistical significance (*p* < 0.10) in the univariate logistic regression model were excluded. A significance level of *p* < 0.10 was chosen to strike a balance between minimizing the risk of excluding potentially important variables and controlling for type I errors (42). In the second step, based on the bio-psycho-social model, three multiple logistic regression models were constructed sequentially: (a) Model 1, which included only biological variables, (b) Model 2, where psychological variables were added to Model 1, and (c) Model 3, in which social variables were included in Model 2. Only variables determined to be significant (*p* < 0.05) in each model were retained for subsequent analysis. Therefore, a stepwise logistic regression modeling approach was employed. Before constructing the multiple logistic regression models in the second step, we used tolerance values and variance inflation factors (VIFs) to assess multicollinearity between the independent variables. The results indicated no evidence of multicollinearity. The log-likelihood ratio test and the Hosmer–Lemeshow test were applied to examine the fit of the multiple logistic regression models.

All the statistical analyses were performed using IBM SPSS Statistics software, version 26 (IBM, Armonk, NY, United States). A significance level of *p* value < 0.05 was considered indicate statistically significant (two-tailed).

## 3 Results

The biological, psychological, and social characteristics of the participants are presented in Table 1 and Supplementary Table S1, both for the total sample and stratified by PA-related injuries group. The study included 1,051 first-year undergraduate students with the following age distribution: 18 years (73.64%), 19 years (23.79%), 20 years and above (2.57%). Among them, 564 individuals (53.66%) were male, 634 individuals (60.32%) had a normal weight, 916 individuals (87.16%) were myopic, and 977 individuals (92.96%) were free of chronic diseases. Additionally, 823 individuals (78.31%) considered themselves to be in good physical fitness, 597 individuals (56.80%) exhibited extroverted personality traits, and 928 individuals (88.30%) did not participate in any sports teams. In terms of health behaviors, participants reported an average of 6.91 h of sedentary time per day (SD = 3.068), an average screen time of 5.51 h daily (SD = 2.680), and an average sleep duration of 7.34 h per day (SD = 0.779). Notably, 544 individuals (51.76%) failed to meet the advised PA levels. Further detailed information concerning the biological, psychological, and social aspects is available in Table 1 and Supplementary Table S1, providing a comprehensive overview of the various variables.

**Table 1:** Descriptive characteristics of the participants in terms of biological and psychological aspects.

Variable	Total (*n* = 1,051)	PA-related injuries (*n* = 296)	Non-PA related injuries (*n* = 755)
**Biological variables**			
**Age**			
18	774 (73.64)	227 (76.69)	547 (72.45)
19	250 (23.79)	64 (21.62)	186 (24.64)
≥20	27 (2.57)	5 (1.69)	22 (2.91)
**Gender**			
Male	487 (46.34)	172 (58.11)	315 (41.72)
Female	564 (53.66)	124 (41.89)	440 (58.28)
**BMI**			
Underweight	268 (25.50)	72 (24.32)	196 (25.96)
Normal weight	634 (60.32)	176 (59.46)	458 (60.66)
Overweight	105 (9.99)	31 (10.47)	74 (9.80)
Obese	44 (4.19)	17 (5.74)	27 (3.58)
**Myopia status**			
No	135 (12.84)	41 (13.85)	94 (12.45)
Yes	916 (87.16)	255 (86.15)	661 (87.55)
**Chronic diseases**			
No	977 (92.96)	266 (89.86)	711 (94.17)
Yes	74 (7.04)	30 (10.14)	44 (5.83)
**Physical fitness**			
Poor	823 (78.31)	234 (79.05)	589 (78.01)
Good	228 (21.69)	62 (20.95)	166 (21.99)
**PA during fatigue**			
Never	284 (27.02)	55 (18.58)	229 (30.33)
Rarely	408 (38.82)	108 (36.49)	300 (39.74)
Sometimes	273 (25.98)	97 (32.77)	176 (23.31)
Frequently	86 (8.18)	36 (12.16)	50 (6.62)
**PA during illness/injury**			
Never	442 (42.06)	96 (32.43)	346 (45.83)
Rarely	433 (41.20)	133 (44.93)	300 (39.74)
Sometimes	141 (13.42)	53 (17.91)	88 (11.66)
Frequently	35 (3.33)	14 (4.73)	21 (2.78)
**Psychological variables**			
**Extroversion**			
Introverted	224 (21.31)	49 (16.55)	175 (23.18)
Neutral	230 (21.88)	69 (23.31)	161 (21.32)
Extroverted	597 (56.80)	178 (60.14)	419 (55.50)
**Neuroticism**			
Absent	199 (18.93)	43 (14.53)	156 (20.66)
Mild	115 (10.94)	28 (9.46)	87 (11.52)
Severe	737 (70.12)	225 (76.01)	512 (67.81)
**Psychoticism**			
Absent	586 (55.76)	147 (49.66)	439 (58.15)
Mild	70 (6.66)	30 (10.14)	40 (5.30)
Severe	395 (37.58)	119 (40.20)	276 (36.56)
**Obsessive/compulsive**			
Absent	675 (64.22)	175 (59.12)	500 (66.23)
Mild	348 (33.11)	110 (37.16)	238 (31.52)
Severe	28 (2.66)	11 (3.72)	17 (2.25)
**Depression**			
Absent	858 (81.64)	228 (77.03)	630 (83.44)
Mild	180 (17.13)	63 (21.28)	117 (15.50)
Severe	13 (1.24)	5 (1.69)	8 (1.06)
**Anxiety**			
Absent	776 (73.83)	204 (68.92)	572 (75.76)
Mild	262 (24.93)	88 (29.73)	174 (23.05)
Severe	13 (1.24)	4 (1.35)	9 (1.19)
**Hostility**			
Absent	940 (89.44)	256 (86.49)	684 (90.60)
Mild	113 (9.80)	36 (12.16)	67 (8.87)
Severe	8 (0.76)	4 (1.35)	4 (0.53)
**Paranoia ideation**			
Absent	867 (82.49)	231 (78.04)	636 (84.24)
Mild	177 (16.84)	62 (20.95)	115 (15.23)
Severe	7 (0.67)	3 (1.01)	4 (0.53)
**Self-protective awareness**			
Poor	49 (4.66)	163 (55.07)	410 (54.30)
Normal	429 (40.82)	118 (39.86)	311 (41.19)
Good	573 (54.52)	15 (5.07)	23 (4.50)

Data are presented as n (percentage). The percentage may not add to 100% due to rounding.

SD, standard deviation; BMI, body mass index; PA, physical activity; PA-related injuries, physical activity-related injuries.

In the past 12 months, 296 university students (28.16%) reported having experienced PA-related injuries. Among them, 159 students (53.72%) experienced PA-related injuries only once, 86 students (29.05%) experienced PA-related injuries twice, and 51 students (17.23%) experienced PA-related injuries three or more times. More than half (67.57%) of the students who were injured reported a mild injury, with only 2.03% describing their injury as severe. 30.41% of the students reported a moderate injury. Furthermore, 44.93% of the injured students received no treatment, 29.39% received physical therapy or rehabilitation, 19.26% received wound disinfection and bandaging, 2.36% received surgery, and 4.05% received other forms of treatment.

**Figure 1:**
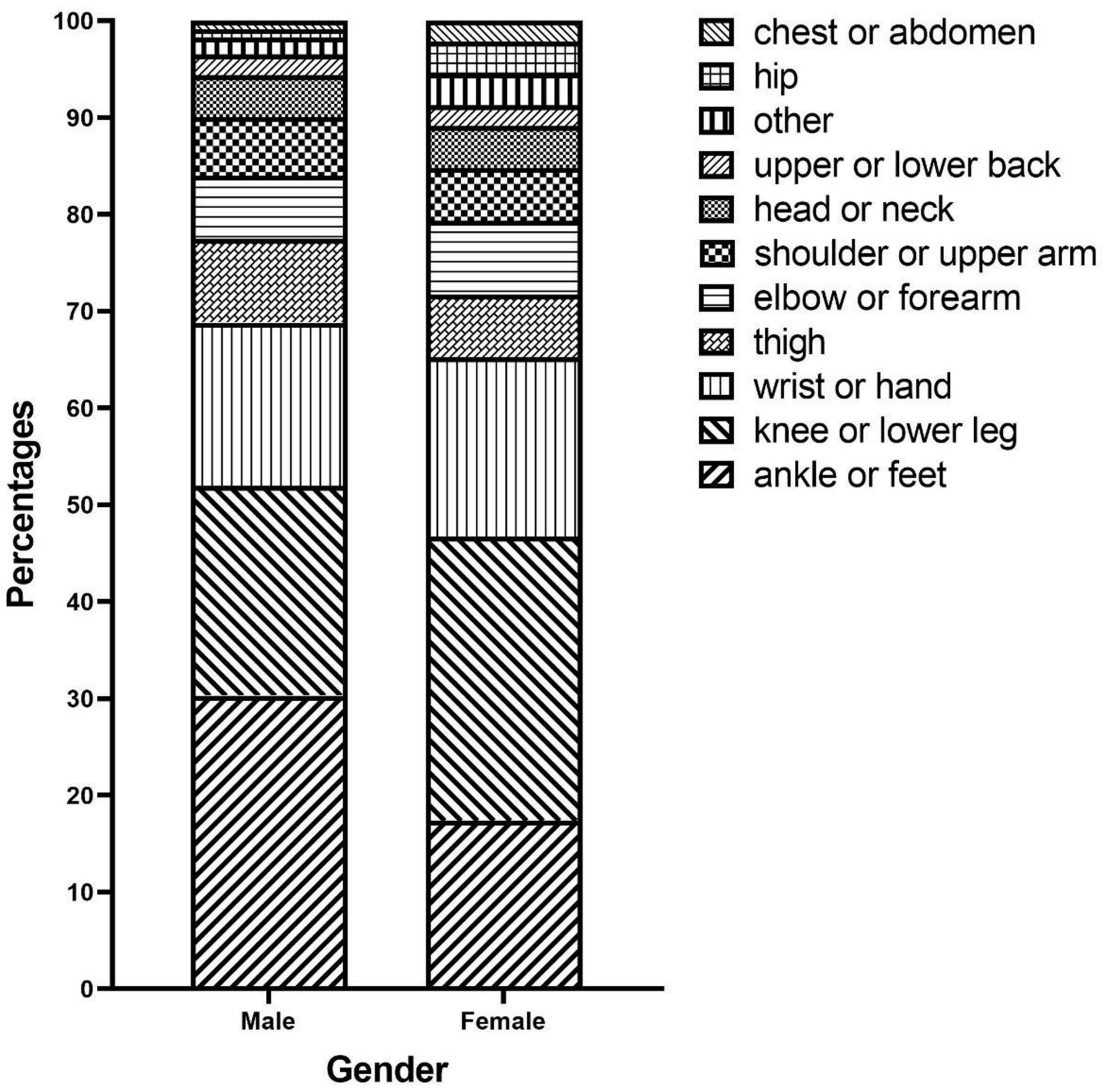
Distribution of injured body parts by gender.

**Figure 2:**
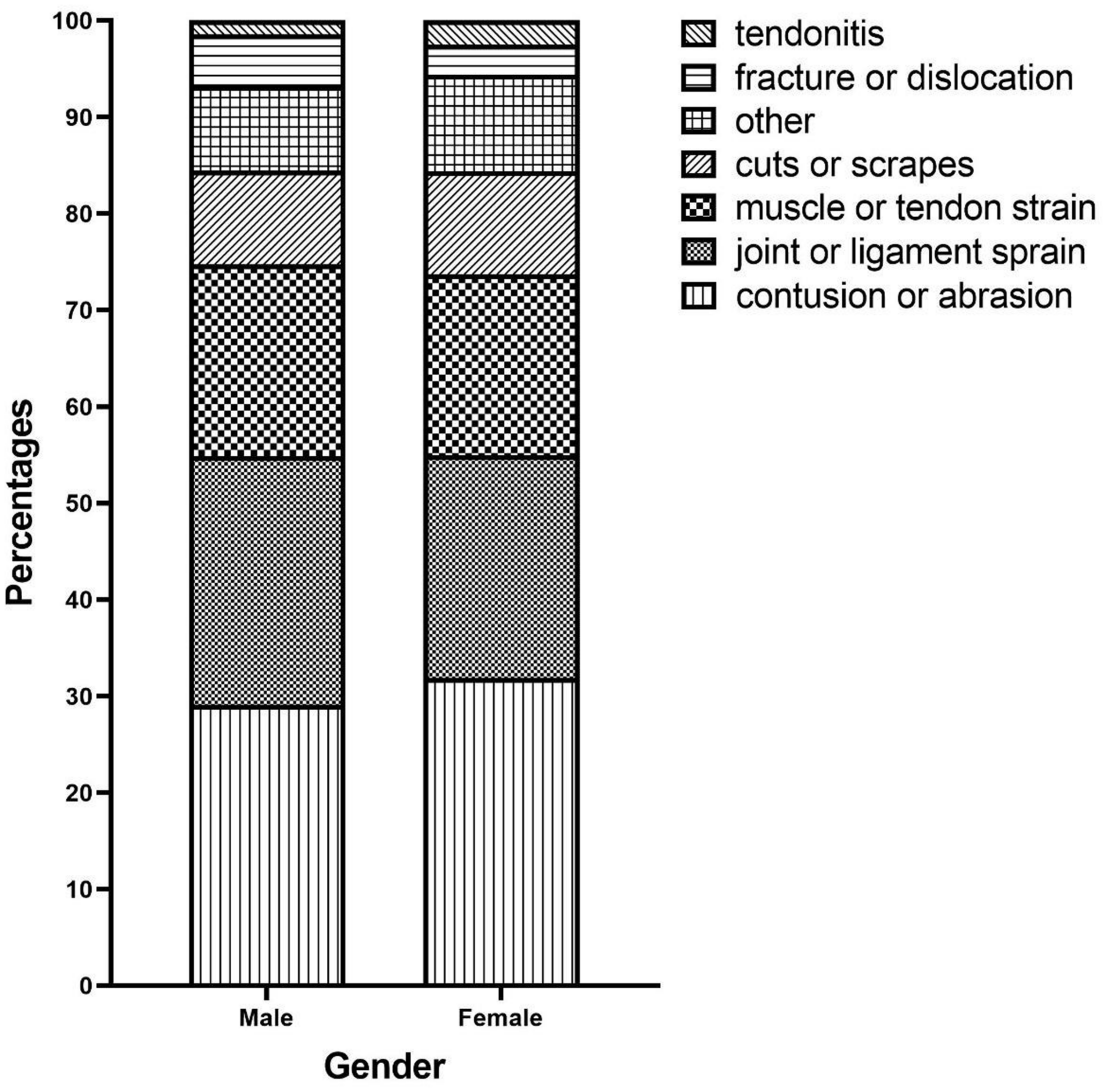
Distribution of injury types by gender.

**Figure 3:**
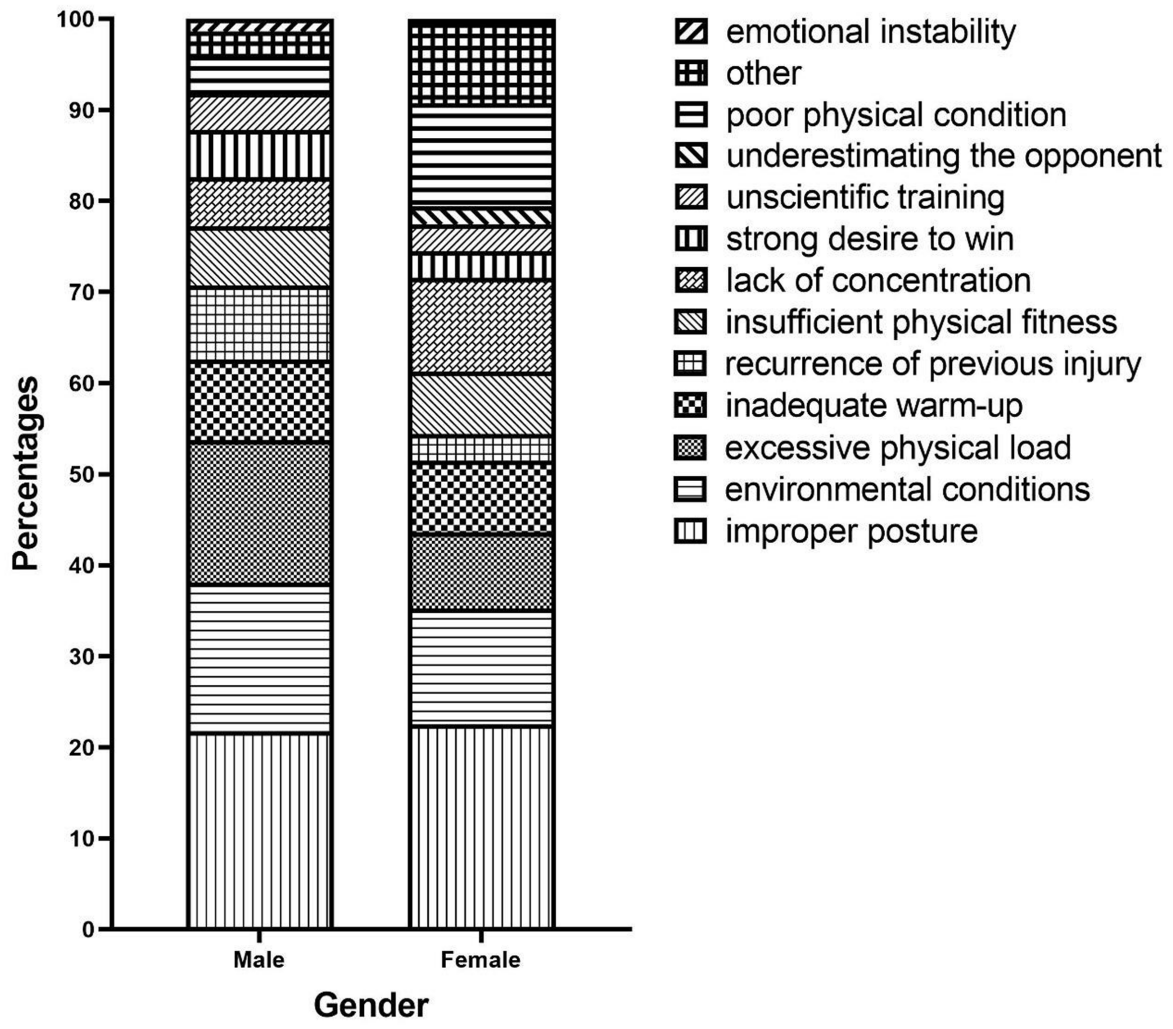
Distribution of injury causes by gender.

Additionally, we documented one or more injured body parts reported by injured students during their latest PA-related injuries incident, resulting in 415 injured body parts. The most frequently injured body parts were the knee or lower leg (25.06%), the ankle or feet (24.58%), and the wrist or hand (17.59%). Similarly, we collected data on injury types reported by injured students, resulting in 366 reported injury types. The most common injury types were contusion or abrasion (30.33%), muscle or tendon strain (24.59%), and joint or ligament sprain (19.40%). In terms of injury causes, injured students reported a total of 498 injury causes, with improper posture (22.09%) being the most common, followed by environmental conditions (14.86%) and excessive physical load (12.56%). The distributions of injured body parts, injury types, and injury causes are visually presented in a stacked bar chart grouped by gender (Figures 1–3). By comparing the differences in injury patterns between genders, we can more accurately understand the characteristics of injuries in each gender.

Univariable regression analysis revealed that 25 out of 34 variables across biological, psychological, and social aspects were significantly associated with PA-related injuries among first-year university students. Variables including age, BMI, myopia, physical fitness, self-protective awareness, sedentary time, screen time, sleep duration, and PA frequency, exceeded the predetermined significance threshold of 0.1 and were subsequently excluded from further analysis in Step 2. In Supplementary Table S2, we present the odds ratios, corresponding confidence intervals, and *p*-values for the significant variables identified in the univariable regression analysis. To comprehensively evaluate the impact of these significant variables on PA-related injuries and identify potential predictive factors, we employed three multiple logistic regression models, as shown in Table 2.

**Table 2:** Multiple logistic regression analysis of PA-related injuries.

	Model 1: biological		Model 2: biological + psychological		Model 3: biological + psychological + social	
	OR (95%CI)	*p* value	OR (95%CI)	*p* value	OR (95%CI)	*p* value
**Biological variables**						
**Gender**						
Male	1 (Ref)		1 (Ref)		1 (Ref)	
Female	0.55 (0.42–0.73)	<0.001**	0.55 (0.41–0.75)	<0.001**	0.67 (0.47–0.94)	0.022*
**Chronic diseases**						
No	1 (Ref)		1 (Ref)		1 (Ref)	
Yes	1.88 (1.14–3.12)	0.014*	1.81 (1.09–3.02)	0.023*	1.70 (0.98–2.93)	0.058
**PA during fatigue**						
Never	1 (Ref)		1 (Ref)		1 (Ref)	
Rarely	1.20 (0.81–1.80)	0.362	1.37 (0.93–2.00)	0.110	0.98 (0.63–1.50)	0.909
Sometimes	1.78 (1.17–2.70)	0.007**	2.06 (1.38–3.07)	<0.001**	1.50 (0.95–2.37)	0.080
Frequently	2.21 (1.24–3.95)	0.007**	2.55 (1.48–4.37)	<0.001**	1.78 (0.96–3.28)	0.066
**PA during illness/injury**						
Never	1 (Ref)		Exc		Exc	
Rarely	1.34 (0.96–1.87)	0.087				
Sometimes	1.55 (1.00–2.41)	0.053				
Frequently	1.36 (0.62–3.00)	0.446				
**Psychological variables**						
**Extroversion**						
Introverted			1 (Ref)		1 (Ref)	
Neutral			1.57 (1.01–2.46)	0.046*	1.46 (0.93–2.32)	0.104
Extroverted			1.66 (1.12–2.44)	0.012*	1.23 (0.82–1.84)	0.323
**Neuroticism**						
Absent			1 (Ref)		1 (Ref)	
Mild			1.18 (0.67–2.07)	0.561	1.07 (0.59–1.92)	0.829
Severe			1.53 (1.01–2.44)	0.045*	1.61 (1.07–2.41)	0.022*
**Psychoticism**						
Absent			1 (Ref)		Exc	
Mild			1.70 (0.99–2.93)	0.056		
Severe			1.05 (0.77–1.44)	0.741		
**Obsessive/compulsive**						
Absent			1 (Ref)		Exc	
Mild			1.04 (0.70–1.54)	0.849		
Severe			2.21 (0.73–6.70)	0.161		
**Depression**						
Absent			1 (Ref)		Exc	
Mild			1.40 (0.81–2.42)	0.227		
Severe			1.54 (0.30–7.97)	0.610		
**Anxiety**						
Absent			1 (Ref)		Exc	
Mild			1.00 (0.62–1.60)	0.984		
Severe			0.32 (0.05–2.03)	0.225		
**Hostility**						
Absent			1 (Ref)		Exc	
Mild			0.963 (0.55–1.69)	0.895		
Severe			1.95 (0.34–10.18)	0.454		
**Paranoia ideation**						
Absent			1 (Ref)		Exc	
Mild			1.03 (0.63–1.69)	0.906		
Severe			0.88 (0.15–5.22)	0.890		
**Social variables**						
**College**						
College of Law					1 (Ref)	
College of Engineering					0.80 (0.46–1.39)	0.425
College of Science					0.85 (0.47–1.55)	0.596
College of Business					0.75 (0.40–1.43)	0.382
College of Liberal Arts					0.47 (0.22–1.00)	0.050
College of Medicine					1.00 (0.56–1.79)	0.996
College of Journalism and Communication					0.46 (0.18–1.16)	0.099
College of Arts and Design					0.48 (0.21–1.13)	0.092
Sports team member						
No					1 (Ref)	
Yes					2.09 (1.34–3.27)	0.001**
**PA duration (per session)**						
≤30 min					1 (Ref)	
0.5 ~ 1 h					1.10 (0.79–1.54)	0.567
≥1 h					1.23 (0.76–1.98)	0.399
**PA level**						
Below					1 (Ref)	
Meet					1.12 (0.81–1.55)	0.511
Above					1.19 (0.62–2.27)	0.603
**Smoking**						
No					1 (Ref)	
Yes					2.06 (0.83–5.12)	0.122
**Alcohol consumption**						
No					1 (Ref)	
Yes					1.22 (0.85–1.76)	0.285
**PA on the wet ground**						
Never					1 (Ref)	
Rarely					1.73 (1.18–2.54)	0.005**
Sometimes					1.56 (0.87–2.82)	0.138
Frequently					1.24 (0.24–6.37)	0.801
**PA on the uneven floor**						
Never					1 (Ref)	
Rarely					1.20 (0.81–1.77)	0.363
Sometimes					1.10 (0.68–1.80)	0.697
Frequently					1.82 (0.71–4.68)	0.212
**PA under insufficient light**						
Never					1 (Ref)	
Rarely					1.07 (0.70–1.64)	0.764
Sometimes					1.09 (0.70–1.72)	0.697
Frequently					1.29 (0.68–2.45)	0.437
**PA during hot weather**						
Never					1 (Ref)	
Rarely					1.01 (0.69–1.47)	0.968
Sometimes					1.56 (0.98–2.49)	0.061
Frequently					0.30 (0.08–1.08)	0.066
**PA during cold weather**						
Never					1 (Ref)	
Rarely					0.82 (0.51–1.32)	0.405
Sometimes					0.88 (0.54–1.42)	0.598
Frequently					1.42 (0.69–2.89)	0.340
**PA during rainy weather**						
Never					1 (Ref)	
Rarely					0.91 (0.64–1.30)	0.611
Sometimes					0.64 (0.34–1.20)	0.165
Frequently					0.83 (0.17–4.12)	0.820
**PA in crowded places**						
Never					1 (Ref)	
Rarely					0.97 (0.68–1.40)	0.883
Sometimes					0.77 (0.40–1.48)	0.425
Frequently					1.00 (0.30–3.32)	0.997
−2 log likehood	1194.244^a^		1174.945^a^		1122.846^a^	
Hosmer–Lemeshow test	0.997		0.904		0.473	

a Indicates a statistical significance in the model fit, with a *p*-value less than 0.001.

*p < 0.05; **p < 0.01.

PA-related injuries, physical activity-related injuries; PA, physical activity; OR, odds ratio; 95%CI, 95% confidence interval; Ref, reference category; Exc, excluded from the model because of the inclusion criteria.

According to the results of Model 3, within the biological factors, female university students had lower odds of PA-related injuries (OR = 0.67, 95% CI: 0.47–0.94, *p* = 0.022) compared to their male counterparts. Within the psychological factors, individuals with high neuroticism showed greater odds of PA-related injuries (OR = 1.61, 95% CI: 1.07–2.41, *p* = 0.022) compared to emotionally stable individuals. Among the social factors, university students who were members of sports teams reported greater odds of PA-related injuries (OR = 2.09, 95% CI: 1.34–3.27, *p* < 0.001) compared to those who were not members of sports teams. Students who reported rarely engaging in PA on the wet ground had greater odds of PA-related injuries (OR = 1.73, 95% CI: 1.18–2.54, *p* = 0.005) compared to those who never engaged in PA on the wet ground.

The fitness of multiple logistic regression models was assessed in this study using both the log-likelihood ratio test and the Hosmer–Lemeshow test. The results from the log-likelihood ratio test showed that all three models developed good fits, with decreasing residuals of variance. Additionally, the results of the Hosmer–Lemeshow test confirmed well-calibrated predictions (*p* > 0.05), implying that the predicted probabilities were close to the observed outcomes.

## 4 Discussion

The present study examined the variables associated with first-year university students’ PA-related injuries across biological, psychological, and social aspects by means of multiple logistic regression analysis. Our findings revealed several significant influences on PA-related injuries among Chinese first-year university students. Notably, our study found that 28.16% of students reported experiencing PA-related injuries at least once in the past 12 months. This high prevalence underscores the importance of understanding PA-related injuries among university students. Gender was associated with PA-related injuries, indicating that females were less likely to experience PARI. In addition, neuroticism, sports team membership, engagement in PA on the wet ground were associated with PA-related injuries. These findings provide valuable insights into the factors influencing PA-related injuries among first-year university students, and may inform interventions and health promotion strategies for this population. For instance, students with a higher risk of PA-related injuries may benefit from additional psychological counseling and support to enhance their coping mechanisms for potential injuries during PA.

As a biological factor, gender remained a consistently strong and independent predictor of PA-related injuries in all multiple logistic regression models, while chronic disease and PA during fatigue were no longer statistically significant in the final model. Gender is a noteworthy associated factor for PA-related injuries, with males exhibiting a higher risk than females, which is consistent with the findings of previous research (6, 7, 18).

The gender difference in injury risk can be explained by biological and behavioral differences. Typically, males have greater muscle strength and aerobic capacity (43), which may lead them to exceed their physical limits, especially during high-intensity activities. Consequently, the substantial stress placed on joints, muscles, and ligaments during PA may increase the risk of injury. Additionally, behavioral differences in PA patterns further contribute to the observed gender difference in PA-related injuries (44, 45). However, a 1-year prospective study concluded that there was no difference in injury incidence density or injury risk between males and females (13). This lack of difference may be attributed to the limited sample size of the prospective study, which may not adequately assess gender differences. Chronic disease was another biological factor examined in our study, and it had no significant impact on PA-related injuries. This lack of effect may be attributed to the binary nature of the chronic disease variable, which dichotomized health conditions without considering potential diversity among the participants’ health conditions. Nevertheless, a study across multiple centers indicated that chronic diseases, considered a binary variable, are a potential determinant of PA-related injuries in females (6). This association might be associated with the overall health status of the study participants, influencing their engagement in PA and potentially increasing their risk of injury during PA.

Moreover, the statistical significance of the association between PA during fatigue and PA-related injuries was absent in Model 3, similar to the chronic disease variable. This finding suggested that the addition of social variables in Model 3 potentially diminished the previously observed association between PA during fatigue and PA-related injuries. The absence of statistical significance in Model 1 for PA during illness or injury might be attributed to the limited participation in PA during illness or injury periods. In addition, our findings suggest non-significant associations between age, BMI, myopia status, and physical fitness and PA-related injuries. These results may be attributed to the distinct characteristics of the study population, along with the influence of social desirability bias. The distinct characteristics might include a specific age distribution, health conditions, or lifestyle habits that differ from the general population, potentially affecting the impact of age, BMI, myopia status, and physical fitness on PA-related injuries. For instance, if the study participants are mostly physically active and health-conscious individuals, they might take more preventive measures to avoid injuries, which could explain the lack of significant associations found. Furthermore, social desirability bias refers to the tendency of individuals to provide socially acceptable responses rather than accurate ones, and may distort reporting on PA, health status, and PA-related injuries. This bias could obscure true associations and impact the interpretation of our results. Consequently, these biological factors should be further investigated using more diverse samples and more precise measurement methods.

In terms of psychological factors, our study identified that eight psychological factors exhibited statistically significant associations with PA-related injuries in the univariate logistic regression analysis, apart from self-protective awareness. Notably, a study reported that middle school students with greater safety awareness appeared to have a lower risk of PA-related injuries (14). The lack of statistical significance in our investigation may be ascribed to the measurement methods employed for self-protective awareness. Furthermore, the inclusion of social factors in multiple regression Model 3 resulted in the exclusion of most psychological variables. Our analysis showed that only neuroticism was significantly associated with PA-related injuries, while other psychological variables, including psychoticism, extroversion, hostility, paranoia ideation, obsessive/compulsive, anxiety, and depression, were not significantly associated with PA-related injuries. This lack of association may be attributed to the complex interplay among these psychological variables and might have been influenced by social factors. For example, these psychological variables may have interdependent associations with PA-related injuries rather than independent associations, indicating that the significance of each variable may be weakened or even diminished. For example, neuroticism is characterized by a disposition to experience anxiety, depression, and other negative emotions (46), and can lead to psychological and physical stress. This stress may negatively impact an individual’s physical condition, which could indirectly influence the risk of PA-related injuries. Previous injury-prediction models, as proposed by Williams and Andersen (47), emphasize the importance of personality traits and stress factors in the occurrence of injuries. A study on athlete injuries identified trait anxiety and stress resulting from negative life events as significant predictors of sports-related injuries (17). Nonetheless, these associations may not represent a direct causal relationship and could be influenced by other factors. Further research should investigate the intricate interplay between psychological and social factors to develop a more comprehensive understanding of the risk related to PA-related injuries.

Among the four associated factors identified in our multiple logistic regression analysis, sports team membership and PA on the wet ground were associated with social aspects. These results demonstrate the impact of social factors on the risk of PA-related injuries. The positive association observed between sports team membership and PA-related injuries indicated a significantly higher risk of PA-related injuries among sports team members. This result is consistent with previous studies that have identified sports team membership as a potential associated factor for PA-related injuries (6, 18, 19, 48), emphasizing the significance of targeted injury prevention measures in the context of sports. However, the absence of statistical significance in the college variable can be attributed to the characteristics of our study participants. First-year university students typically have a general education curriculum after admission, which might contribute to limited distinction among students across different colleges. Furthermore, the results of this study revealed no statistically significant difference in the risk of PA-related injuries between smokers and non-smokers. This lack of significance may be attributed to the relatively limited effects of smoking on pulmonary function and cardiovascular health (49), which may not have been substantial enough to influence the risk of PA-related injuries, particularly among first-year university students whose smoking duration may be short. Notably, there was no statistically significant association between alcohol consumption and PA-related injuries, suggesting that the influence of alcohol on injury risk during PA may only manifest when alcohol is consumed concurrently with engaging in PA.

Activity-related behaviors can directly affect injury risk and serve as risk factors, while also potentially influencing risk factors and injury mechanisms, thus indirectly affecting injury risk (50). Previous studies have indicated that sedentary time (6, 9), screen time (10), sleep duration (6, 21–23), and PA (11, 12, 48, 51), are associated with PA-related injuries. Our study revealed that among these variables, only PA duration and PA level had statistical significance according to univariate logistic regression analysis. However, these significant associations did not persist in the subsequent multiple logistic regression analysis. This result could be attributed to the characteristics of our study participants, who were first-year students just starting their academic journey. During this period, students display relatively similar patterns of activity behavior as they adapt to the new university environment and academic demands (52). Moreover, our study did not distinguish between various intensity levels (low, moderate, vigorous) and specific types of PA, which could result in the non-significance of PA duration and level in the multiple regression analysis. Our findings demonstrate both similarities and disparities compared to prior studies on the influence of environmental variables. Specifically, PA on the wet ground maintained statistical significance in the multiple regression analysis, which is consistent with the findings of prior studies (10, 14). However, an increased frequency of PA on the wet ground did not demonstrate a significant correlation with an increased risk of PA-related injuries in our study. Furthermore, while PA during cold weather showed significance in univariate analysis, it was not significantly associated with PA-related injuries among Chinese university students in the multiple regression analysis. It’s noteworthy that previous studies also identified the statistical significance of this factor in univariate analysis (10, 14). The disparities observed in our findings compared to prior studies may be attributed to specific characteristics of the participants and variations in study methodologies, such as sample size and data collection methods, contributing to disparities in statistical significance and risk associations.

These results suggest that biological, psychological, and social aspects are all potential contributors to PA-related injuries among university students. However, it is crucial to acknowledge the study’s limitations in accurately interpreting these factors. First, the cross-sectional design restricts the establishment of causal relationships, given its limitations in addressing reverse causality or bidirectional relationships. In addition, this design introduces the potential for recall bias, unmeasured confounders, and residual confounding. Second, the reliance on self-report questionnaires in our study introduces potential biases, such as recall bias and social desirability bias. Consequently, a cautious interpretation of these findings is essential. Future research should consider more objective measures to increase the data accuracy. Third, our study lacked detailed information on family background, sleep quality, and specific details of PA, such as intensity and type. This limitation underscores the need for future research to collect more comprehensive data on these variables, which may explain the observed associations more accurately. Finally, this study only included a sample of individuals from Shantou University, potentially restricting the generalizability of the results to other populations. Due to our limited sample size, these findings warrant further investigation to ascertain their robustness and generalizability.

Despite its limitations, our study offers valuable insight into the understanding of PA-related injuries among university students. The comprehensive examination of biological, psychological, and social factors establishes a basis for future research and will facilitate the development of comprehensive prevention measures that consider bio-psycho-social factors, leading to more effective and comprehensive prevention of PA-related injuries. Moreover, our study participants are first-year university students entering adulthood, and their heightened receptivity and active engagement in PA offer a practical opportunity to advocate for PA-related injuries prevention, especially compared to older adults who will face academic and professional demands in the future. During this crucial transitional period, implementing preventive measures not only ensures immediate effects but also fosters lasting benefits in the later stages of adult life, aligning with public health initiatives aimed at enhancing the overall health of the population.

## 5 Conclusion

This study is the first to explore the factors associated with PA-related injuries among Chinese first-year university students within a bio-psycho-social framework. Our findings highlight the significant impact of multiple factors in biological, psychological, and social aspects on university students’ PA-related injuries and emphasize the importance of a comprehensive approach to understanding and preventing PA-related injuries. There is a need to identify high-risk individuals based on their physiological and psychological characteristics and implement measures aimed at modifiable risk factors to reduce the occurrence of PA-related injuries. Future research could consider longitudinal designs or intervention studies, contributing to the success of PA-related injuries prevention and PA promotion among university students.

## Data availability statement

The raw data supporting the conclusions of this article will be made available by the authors, without undue reservation.

## Ethics statement

The studies involving humans were approved by Shantou University Medical College Ethics Committee. The studies were conducted in accordance with the local legislation and institutional requirements. The participants provided their written informed consent to participate in this study.

## Author contributions

LX: Data curation, Visualization, Writing – original draft, Writing – review & editing. SC: Investigation, Writing – review & editing. DG: Investigation, Writing – review & editing. YF: Investigation, Writing – review & editing. LL: Project administration, Supervision, Writing – review & editing.
