# Supplementary material for: The associated factors for physical activity-related injuries among first-year university students in southern China from a biopsychosocial perspective
Source: Front Public Health. 2024 Apr 2;12:1369583. doi: 10.3389/fpubh.2024.1369583 (PMC11018952; doi:10.3389/fpubh.2024.1369583)
Supplement: Supplementary file 1 [file Table_1.docx]

Supplementary Table 1. Descriptive characteristics of the participants in social aspects

| **Variable** | **Total (n=1051)** | **PA-related injuries (n=296)** | **Non-PA related injuries (n=755)** |
| --- | --- | --- | --- |
| **Social variables** |  |  |  |
| College |  |  |  |
| College of Law | 97 (9.23) | 28 (9.46) | 69 (9.14) |
| College of Engineering | 285 (27.12) | 89 (30.07) | 196 (25.96) |
| College of Science | 165 (15.70) | 53 (17.91) | 112 (14.83) |
| College of Business | 131 (12.46) | 29 (9.80) | 102 (13.51) |
| College of Liberal Arts | 89 (8.47) | 14 (4.73) | 75 (9.93) |
| College of Medicine | 179 (17.03) | 62 (20.95) | 117 (15.50) |
| College of Journalism and Communication | 46 (4.38) | 8 (2.70) | 38 (5.03) |
| College of Arts and Design | 59 (5.61) | 13 (4.39) | 46 (6.09) |
| Sports team member |  |  |  |
| No | 123 (11.70) | 239 (80.74) | 689 (91.26) |
| Yes | 928 (88.30) | 57 (19.26) | 66 (8.74) |
| Sedentary time (mean ± SD, h/day) | 6.91±3.068 | 6.77±3.111 | 6.97±3.050 |
| Sedentary behavior |  |  |  |
| Low (<4h/day) | 135 (12.84) | 42 (14.19) | 93 (12.32) |
| Moderate (4~8h/day) | 467 (44.43) | 133 (44.93) | 334 (44.24) |
| High (≥8h/day) | 449 (42.72) | 121 (40.88) | 328 (43.44) |
| Screen time (mean ± SD, h/day) | 5.51±2.680 | 5.90±2.726 | 5.78±2.662 |
| Screen use behavior |  |  |  |
| Low (<2h/day) | 16 (1.52) | 6 (2.03) | 10 (1.32) |
| Moderate (2~4h/day) | 166 (15.79) | 42 (14.19) | 124 (16.42) |
| High (≥4h/day) | 869 (82.68) | 248 (83.78) | 621 (82.25) |
| Sleep duration (mean ± SD, h/day) | 7.34±0.779 | 7.30±0.884 | 7.36±0.733 |
| Sleep behavior |  |  |  |
| Insufficient (<7h/day) | 99 (9.42) | 34 (11.49) | 65 (8.61) |
| Adequate (7~9h/day) | 904 (86.01) | 248 (83.78) | 656 (86.89) |
| Excessive (≥9h/day) | 48 (4.57) | 14 (4.73) | 34 (4.50) |
| PA frequency (per week) |  |  |  |
| Never | 18 (1.71) | 4 (1.35) | 14 (1.85) |
| Sometimes (≤2 times) | 701 (66.70) | 170 (57.43) | 531 (70.33) |
| Often (3~5 times) | 265 (25.21) | 105 (35.47) | 160 (21.19) |
| Always (≥6 times) | 67 (6.37) | 17 (5.74) | 50 (6.62) |
| PA duration (per session) |  |  |  |
| ≤30 min/time | 562 (53.47) | 137 (46.28) | 425 (56.29) |
| 0.5~1 h | 356 (33.87) | 109 (36.82) | 247 (32.72) |
| ≥1 h | 133 (1265) | 50 (16.89) | 83 (10.99) |
| PA level |  |  |  |
| Below | 544 (51.76) | 132 (44.59) | 412 (54.57) |
| Meet | 441 (41.96) | 134 (45.27) | 307 (40.66) |
| Above | 66 (6.28) | 30 (10.14) | 36 (4.77) |
| Smoking |  |  |  |
| No | 1025 (97.53) | 281 (94.93) | 744 (98.54) |
| Yes | 26 (2.47) | 15 (5.07) | 11 (1.46) |
| Alcohol consumption |  |  |  |
| No | 836 (79.54) | 215 (72.64) | 621 (82.25) |
| Yes | 215 (20.46) | 81 (27.36) | 134 (17.75) |
| PA on the wet ground |  |  |  |
| Never | 532 (50.62) | 111 (37.50) | 421 (55.76) |
| Rarely | 404 (38.44) | 143 (48.31) | 261 (34.57) |
| Sometimes | 105 (9.99) | 38 (12.84) | 67 (8.87) |
| Frequently | 10 (0.95) | 4 (1.35) | 6 (0.79) |
| PA on the uneven floor |  |  |  |
| Never | 420 (39.96) | 93 (31.42) | 327 (43.31) |
| Rarely | 415 (39.49) | 128 (43.24) | 287 (38.01) |
| Sometimes | 188 (17.89) | 62 (20.95) | 126 (16.69) |
| Frequently | 28 (2.66) | 13 (4.39) | 15 (1.99) |
| PA under insufficient light |  |  |  |
| Never | 260 (24.74) | 61 (20.61) | 199 (26.36) |
| Rarely | 406 (38.63) | 117 (39.53) | 289 (38.28) |
| Sometimes | 308 (29.31) | 91 (30.74) | 217 (28.74) |
| Frequently | 77 (7.33) | 27 (9.12) | 50 (6.62) |
| PA during hot weather |  |  |  |
| Never | 425 (40.44) | 96 (32.43) | 329 (43.58) |
| Rarely | 429 (40.82) | 125 (42.23) | 304 (40.26) |
| Sometimes | 174 (16.56) | 71 (23.99) | 103 (13.64) |
| Frequently | 23 (2.19) | 4 (1.35) | 19 (2.52) |
| PA during cold weather |  |  |  |
| Never | 200 (19.03) | 43 (14.53) | 157 (20.79) |
| Rarely | 376 (35.78) | 95 (32.09) | 281 (37.22) |
| Sometimes | 410 (39.01) | 131 (44.26) | 279 (36.95) |
| Frequently | 65 (6.18) | 27 (9.12) | 38 (5.03) |
| PA during rainy weather |  |  |  |
| Never | 600 (57.09) | 153 (51.69) | 447 (59.21) |
| Rarely | 357 (33.97) | 116 (39.19) | 241 (31.92) |
| Sometimes | 84 (7.99) | 23 (7.77) | 61 (8.08) |
| Frequently | 10 (0.95) | 4 (1.35) | 6 (0.79) |
| PA in crowded places |  |  |  |
| Never | 635 (60.42) | 158 (53.38) | 477 (63.18) |
| Rarely | 323 (30.73) | 107 (36.15) | 216 (28.61) |
| Sometimes | 75 (7.14) | 25 (8.45) | 50 (6.62) |
| Frequently | 18 (1.71) | 6 (2.03) | 12 (1.59) |

Notes: Data are presented as n (percentage). The percentage may not add to 100% due to rounding.

Abbreviations: PA = physical activity; PA-related injuries = physical activity related injuries.

Supplementary Table 2. Univariable logistic regression analysis of PA-related injuries

|  | *OR* (95%*CI*) | *P* value |
| --- | --- | --- |
| **Biological variables** |  |  |
| Age |  |  |
| 18 | 1 (Ref) |  |
| 19 | 1.83 (0.68-4.88) | 0.230 |
| ≥20 | 1.51 (0.55-4.16) | 0.422 |
| Gender |  |  |
| Male | 1 (Ref) |  |
| Female | 0.52 (0.39-0.68) | <0.001** |
| BMI |  |  |
| Underweight | 1 (Ref) |  |
| Normal weight | 1.05 (0.76-1.44) | 0.783 |
| Overweight | 1.14 (0.69-1.88) | 0.606 |
| Obese | 1.71 (0.88-3.33) | 0.112 |
| Myopia status |  |  |
| No | 1 (Ref) |  |
| Yes | 0.88 (0.60-1.31) | 0.542 |
| Chronic diseases |  |  |
| No | 1 (Ref) |  |
| Yes | 1.82 (1.12-2.96) | 0.015* |
| Physical fitness |  |  |
| Poor | 1 (Ref) |  |
| Good | 0.94 (0.68-1.31) | 0.713 |
| PA during fatigue |  |  |
| Never | 1 (Ref) |  |
| Rarely | 1.50 (1.04-2.16) | 0.031* |
| Sometimes | 2.30 (1.56-3.37) | <0.001** |
| Frequently | 3.00 (1.78-5.04) | <0.001** |
| PA during illness/injury |  |  |
| Never | 1 (Ref) |  |
| Rarely | 1.60 (1.18-2.17) | 0.003** |
| Sometimes | 2.17 (1.44-3.27) | <0.001** |
| Frequently | 2.40 (1.18-4.90) | 0.016* |
| **Psychological variables** |  |  |
| Extroversion |  |  |
| Introverted | 1 (Ref) |  |
| Neutral | 1.53 (1.00-2.34) | 0.049* |
| Extroverted | 1.52 (1.06-2.18) | 0.024* |
| Neuroticism |  |  |
| Absent | 1 (Ref) |  |
| Mild | 1.17 (0.68-2.01) | 0.576 |
| Severe | 1.59 (1.10-2.31) | 0.014* |
| Psychoticism |  |  |
| Absent | 1 (Ref) |  |
| Mild | 2.24 (1.35-3.73) | 0.002** |
| Severe | 1.29 (0.97-1.71) | 0.082 |
| Obsessive/compulsive |  |  |
| Absent | 1 (Ref) |  |
| Mild | 1.32 (0.99-1.75) | 0.055 |
| Severe | 1.85 (0.85-4.02) | 0.121 |
| Depression |  |  |
| Absent | 1 (Ref) |  |
| Mild | 1.49 (1.06-2.09) | 0.023* |
| Severe | 1.73 (0.56-5.33) | 0.342 |
| Anxiety |  |  |
| Absent | 1 (Ref) |  |
| Mild | 1.42 (1.05-1.92) | 0.023* |
| Severe | 1.25 (0.38-4.09) | 0.717 |
| Hostility |  |  |
| Absent | 1 (Ref) |  |
| Mild | 1.44 (0.93-2.21) | 0.099 |
| Severe | 2.67 (0.66-10.76) | 0.167 |
| Paranoia ideation |  |  |
| Absent | 1 (Ref) |  |
| Mild | 1.48 (1.05-2.09) | 0.024* |
| Severe | 2.07 (0.46-9.30) | 0.345 |
| Self-protective awareness |  |  |
| Poor | 1 (Ref) |  |
| Normal | 0.86 (0.45-1.64) | 0.646 |
| Good | 0.90 (0.48-1.70) | 0.748 |
| **Social variables** |  |  |
| College |  |  |
| College of Law | 1 (Ref) |  |
| College of Engineering | 1.12 (0.68-1.86) | 0.663 |
| College of Science | 1.17 (0.68-2.02) | 0.582 |
| College of Business | 0.70 (0.38-1.28) | 0.247 |
| College of Liberal Arts | 0.46 (0.22-0.95) | 0.035* |
| College of Medicine | 1.31 (0.76-2.23) | 0.329 |
| College of Journalism and Communication | 0.52 (0.22-1.25) | 0.144 |
| College of Arts and Design | 0.70 (0.33-1.48) | 0.348 |
| Sports team member |  |  |
| No | 1 (Ref) |  |
| Yes | 2.49 (1.70-3.65) | <0.001** |
| Sedentary behavior |  |  |
| Low (<4h/day) | 1 (Ref) |  |
| Moderate (4~8h/day) | 0.88 (0.58-1.34) | 0.553 |
| High (≥8h/day) | 0.82 (0.54-1.24) | 0.345 |
| Screen use behavior |  |  |
| Low (<2h/day) | 1 (Ref) |  |
| Moderate (2~4h/day) | 0.57 (0.19-1.65) | 0.295 |
| High (≥4h/day) | 0.67 (0.24-1.85) | 0.435 |
| Sleep behavior |  |  |
| Insufficient (<7h/day) | 1 (Ref) |  |
| Adequate (7~9h/day) | 0.72 (0.47-1.12) | 0.148 |
| Excessive (≥9h/day) | 0.79 (0.37-1.66) | 0.531 |
| PA frequency (per week) |  |  |
| Never | 1 (Ref) |  |
| Sometimes (≤2 times) | 1.12 (0.36-3.45) | 0.843 |
| Often (3~5 times) | 2.30 (0.74-7.17) | 0.152 |
| Always (≥6 times) | 1.19 (0.34-4.11) | 0.783 |
| PA duration (per session) |  |  |
| ≤30 min | 1 (Ref) |  |
| 0.5~1 h | 1.37 (1.02-1.84) | 0.038* |
| ≥1 h | 1.87 (1.25-2.79) | 0.002** |
| PA level |  |  |
| Below | 1 (Ref) |  |
| Meet | 1.36 (1.03-1.81) | 0.032* |
| Above | 2.60 (1.54-4.39) | <0.001** |
| Smoking |  |  |
| No | 1 (Ref) |  |
| Yes | 3.16 (1,64-7.96) | 0.001** |
| Alcohol consumption |  |  |
| No | 1 (Ref) |  |
| Yes | 1.75 (1.27-2.40) | <0.001** |
| PA on the wet ground |  |  |
| Never | 1 (Ref) |  |
| Rarely | 2.08 (1.55-2.78) | <0.001** |
| Sometimes | 2.15 (1.37-3.37) | <0.001** |
| Frequently | 2.53 (0.70-9.12) | 0.156 |
| PA on the uneven floor |  |  |
| Never | 1 (Ref) |  |
| Rarely | 1.57 (1.15-2.14) | 0.005** |
| Sometimes | 1.73 (1.18-2.53) | 0.005** |
| Frequently | 3.05 (1.40-6.63) | 0.005** |
| PA under insufficient light |  |  |
| Never | 1 (Ref) |  |
| Rarely | 1.32 (0.92-1.89) | 0.128 |
| Sometimes | 1.37 (0.94-2.00) | 0.103 |
| Frequently | 1.76 (1.02-3.05) | 0.043* |
| PA during hot weather |  |  |
| Never | 1 (Ref) |  |
| Rarely | 1.41 (1.04-1.92) | 0.029* |
| Sometimes | 2.36 (1.62-3.45) | <0.001** |
| Frequently | 0.72 (0.24-2.17) | 0.561 |
| PA during cold weather |  |  |
| Never | 1 (Ref) |  |
| Rarely | 1.23 (0.82-1.86) | 0.314 |
| Sometimes | 1.71 (1.15-2.55) | 0.008** |
| Frequently | 2.59 (1.43-4.72) | 0.002** |
| PA during rainy weather |  |  |
| Never | 1 (Ref) |  |
| Rarely | 1.41 (1.06-1.88) | 0.020* |
| Sometimes | 1.10 (0.66-1.84) | 0.712 |
| Frequently | 1.95 (0.54-6.99) | 0.307 |
| PA in crowded places |  |  |
| Never | 1 (Ref) |  |
| Rarely | 1.50 (1.12-2.01) | 0.007** |
| Sometimes | 1.51 (0.90-2.52) | 0.115 |
| Frequently | 1.51 (0.56-4.09) | 0.418 |

Note: Variables that did not show statistical significance (*p*<0.10) in the univariate logistic regression model were excluded in the multiple logistic regression models.

* *p* < 0.05; ** *p* < 0.01.

Abbreviations: PA-related injuries = physical activity-related injuries; *OR* = odds ratio; 95%*CI* =95% confidence interval; PA= physical activity; SD = standard deviation; BMI= body mass index; Ref: reference category.
